# Dose optimization of breast balloon brachytherapy using a stepping Ir192 HDR source

**DOI:** 10.1120/jacmp.v10i1.2903

**Published:** 2009-02-03

**Authors:** Chang Hyun Choi, Sung‐Joon Ye, E. Ishmael Parsai, Sui Shen, Ruby Meredith, Ivan A. Brezovich, Roger Ove

**Affiliations:** ^1^ Radiation Oncology Seoul National University College of Medicine Seoul Korea; ^2^ Department of Radiation Oncology Medical University of Ohio Toledo Ohio U.S.A.; ^3^ Department of Radiation Oncology University of Alabama College of Medicine Birmingham Alabama U.S.A.; ^4^ Department of Radiation Oncology, Brody School of Medicine East Carolina University Greenville North Carolina U.S.A.

**Keywords:** breast balloon brachytherapy, dose optimization, PTV, HDR

## Abstract

To develop dose optimization schemes of breast balloon brachytherapy using a stepping of Ir192 HDR source.

There is a considerable underdosage (11%–13%) of PTV due to anisotropy of a stationary source in breast balloon brachytherapy. We improved the PTV coverage by varying multiple dwell positions and weights. We assumed that the diameter of spherical balloons varied from 4.0 cm to 5.0 cm, that the PTV was a 1‐cm thick spherical shell over the balloon (reduced by the small portion occupied by the catheter path), and that the number of dwell positions varied from 2 to 13 with 0.25‐cm steps, oriented symmetrically with respect to the balloon center. By assuming that the perfect PTV coverage can be achieved by spherical dose distributions from an isotropic source, we developed an optimization program to minimize two objective functions defined as: (1) the number of PTV‐voxels having more than 10% difference between optimized doses and spherical doses, and (2) the difference between optimized doses and spherical doses per PTV‐voxel.

The optimal PTV coverage occurred when applying 8–11 dwell positions with weights determined by the optimization scheme. Since the optimization yields ellipsoidal isodose distributions along the catheter, there is relative skin sparing for cases with source movement approximately tangent to the skin. We also verified the optimization in CT‐based treatment planning systems.

Our volumetric dose optimization for PTV coverage showed close agreement to linear or multiple‐points optimization results from the literature. The optimization scheme provides a simple and practical solution applicable to the clinic.

PACS number: 87.55.de

## I. INTRODUCTION

Breast balloon brachytherapy using a Ir192 high dose rate (HDR) source as a part of breast conserving therapy is now widely used in the treatment of Stage I and II breast cancer. This is due to its ease of use, short learning curve, and a requirement of only one interstitial path through the breast skin.^(1)^ The rationale for this new modality (called “accelerated partial breast irradiation”) is that it eliminates residual foci of tumor near the surgical bed by delivering adequate doses in one week, while reducing doses to normal tissues. This modality is supported by early studies^(^ ^2^ ^–^ ^4^ ^)^ that show approximately 80% of recurrences after lumpectomy occur near the original tumor site. Skin erythema shown in early clinical trials^(^ ^5^ ^,^ ^6^ ^)^ limits its application to patients with a skin depth of >7mm from the balloon surface.

The current dosimetry of this modality is simple, with one source position in the middle of the balloon catheter. However, it is known that part of the planning target volume (PTV) is underdosed due to anisotropy of the source.^(^ ^7^ ^–^ ^9^ ^)^ Recent Monte Carlo studies^(^ ^9^ ^–^ ^11^ ^)^ have shown that doses to the PTV and the skin in the early trials have been overestimated by neglecting the lack of scatter and the attenuation by contrast media in the balloon. This presented two issues: that skin erythema seen in the early trials occurred at lower doses than previously thought, and that the PTV below the skin and along the source axis may not be adequately treated.^(11)^ On the other hand, recent studies^(^ ^8^ ^,^ ^12^ ^)^ have shown that dose optimization by using multiple dwell positions along the balloon catheter can produce better isodose surfaces enclosing the PTV. In these studies, optimization was usually performed by varying the weights of multiple dwell positions to deliver the prescription dose to a few points located 1 cm from the balloon surface. Astrahan et al.^(8)^ included points of interest on the skin (in the case of <1cm depth from the balloon surface) and on the distal PTV boundary as dose constraints in their optimization of multiple dwell times. Although conformal plans optimized by using multiple dwell positions and dwell times are suggested by a recent research protocol (NSABP B39/RTOG 0413), in clinical practice most cases are treated with a stationary source (i.e. a single dwell position).

In the present study, we further developed the optimization schemes for two types of commercially available Ir192 HDR sources and three different diameters of spherical balloons to cover the sizes of balloons in common clinical use. In contrast to the optimization constrained to a few points of interest (described in the literature, Ref. 8), our volumetric optimization included the entire PTV and the skin surface voxels. As shown in optimization for external beam radiotherapy, our optimization was systematically performed by identifying three components: variables, objective functions, and modeling.

## II. MATERIALS AND METHODS

### A. Balloon Catheters and 192Ir HDR Systems

**Figure 1:**
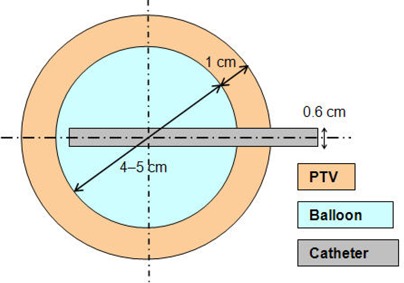
Schematic diagram of the balloon, PTV, and catheter geometry used for optimization.

A balloon catheter (MammoSite RTS; Cytyc, Marlborough, MA) has been developed as a means of delivering partial breast radiotherapy. The device consists of a catheter shaft of approximately 6 mm diameter and 15 cm length with a silicone balloon. The shaft contains a small inflation channel and a larger central treatment channel for passage of an HDR source. An injection port is attached to the inflation channel, and a Luer fitting is attached to the treatment channel. An adapter is provided to connect with a remote after‐loading device. The balloon is inflated with sterile saline to a diameter of 4.0–5.0 cm (30–70cm^3^ inflation volume) in a lumpectomy cavity, and is nearly spherical in shape^(12)^ (Fig. 1).

Two commercial Ir192 HDR sources have been investigated in this study. The microSelectron HDR system (Nucletron Corp., Veenendaal, The Netherlands) uses a 0.65‐mm‐diameter, 3.6‐mm‐long iridium wire, encapsulated in a stainless steel tube of 0.9 mm outer diameter and 0.65 mm inner diameter. The Varisource HDR system (Varian Medical Systems, Inc., Palo Alto, CA) has a source consisting of two pieces of 0.34‐mm‐diameter, 2.5‐mm‐long iridium wires (total active length=5.0mm), encapsulated in a titanium/nickel tube of 0.59 mm outer diameter and 0.34 mm inner diameter. The commercial treatment planning systems of microSelectron and Varisource are Plato (Plato™, Nucletron Corp., Veenendaal, The Netherlands) and BrachyVision (BrachyVision™, Varian Medical Systems BrachyTherapy, Charlottesville, VA), respectively, which were used to retrospectively verify our optimization scheme in CT‐based treatment plans.

### Volume Optimization

**Figure 2:**
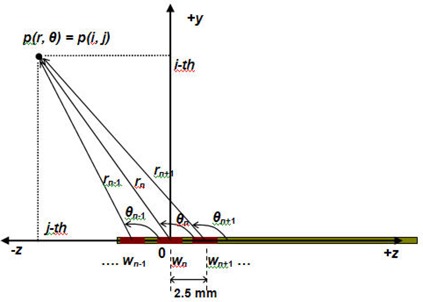
Coordinates employed for TG‐43 dose calculations and schematic diagrams of multiple dwell positions for optimization.

The optimization aimed to provide the prescription dose to the entire PTV and to spare the skin if the minimum skin‐balloon distance was less than 1 cm. The region of interest for our investigation spanned from r=0.25to10.0cm and from θ=0° to 180° in the Polar coordinate (Figs. 1 and 2), which includes the balloon, PTV, skin, and normal breast tissue. As in the early studies,^(7)^ ^,^ ^(8)^ ^,^ ^(12)^ the PTV is a spherical shell occupying the space between the balloon surface and a concentric sphere having a radius of 1 cm plus the balloon radius (Fig. 1). The small cylindrical volume (0.6 cm diameter) occupied by the catheter is not considered part of the PTV. Three balloons, having diameters of 4.0, 4.5, and 5.0 cm, were evaluated.

Because of the spherical symmetry of the PTV, spherical dose distributions would be ideal. Such dose distributions from a linear source can be calculated by eliminating the anisotropic characteristics from the AAPM TG‐43 dose distributions. The optimization was then performed by matching the PTV doses calculated by the AAPM TG‐43 formalism to the ideal spherical doses as much as possible. For the purpose of illustration, the source movement was chosen to be approximately tangent to the skin, as is typically the case in clinical practice. Subsequently, voxels analyzed in the case of skin sparing optimization were located in the anterior portion of a spherical shell encompassed by two spheres of radius $r_{1}$ and $r_{2}$, where $r_{1}=\text{balloon radius plus}1.0625\;\text{cm}$, and $r_{2}=\text{balloon radius plus}0.9375\;\text{cm}$. During the optimization, ideal spherical doses to these voxels were constrained to 90% of the prescription dose for skin sparing.

Like in other optimization procedures for radiotherapy, the optimization consisted of three components: variables, objective functions, and modeling. Dose produced by the actual sources were computed according to the AAPM TG‐43 formalism (i.e. modeling). The dosimetric data of the two sources required by the AAPM TG‐43 method were taken from those noted in Daskalov et al.^(13)^ and Angelopoulos et al.^(14)^ The dose calculation grid (voxel resolution) was 0.125 cm for both axial and transaxial directions. The TG‐43 parameters given in the literature were linearly interpolated at this resolution (Cubic spine). Because of the physical length of the balloon catheters, the maximum number of dwell positions had to be limited to 13 with a 0.25 cm step‐wise movement. Two objective functions (optimizers) were developed: (1) one to minimize the number of PTV‐voxels having more than 10% difference between optimized doses and spherical doses, Minimize Number of Voxels (MNV) ‐); (2) the other to minimize the difference between optimized doses and spherical doses, averaged over all PTV‐voxels, Minimize Difference of Doses (MDD). Unlike external beam irradiation, variables (degrees of freedom) to optimize dose distributions were limited to the number of dwell positions and weight distributions (dwell times).

**Figure 3:**
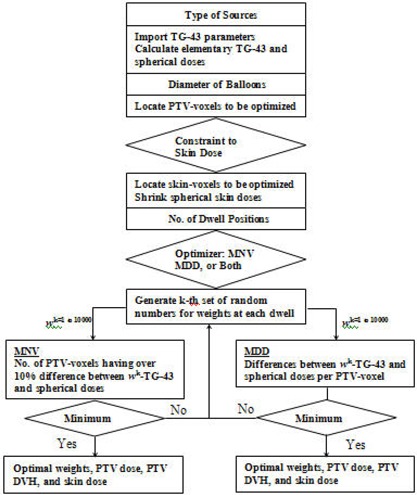
Flow‐chart of optimization procedure.

The optimization was performed by varying the number of dwell positions as well as the weights of each dwell position. Weights at each dwell position were uniformly sampled from a pseudo random space of 0 to 1.0. The number of random seeds at each dwell position was $1.0\times 10^{4}$. Thus, f or a given number of dwell positions N, 10^4^ sets of weights were independently generated (from $N\times 10^{4}$ of pseudo random numbers) and then the sum of each set was normalized to 1.0 for calculation of dwell times. Among $1.0\times 10^{4}$ sets of weights, an optimal set of weights was determined by searching the entire space to minimize the objective functions (either MNV or MDD). Finally, the optimal number of dwell positions, optimal weights, isodose curves, DVH (dose volume histogram) for PTV, and skin dose were determined. Figure 3 shows the schematic diagram (flow chart) of the optimization procedure. The optimization program has been developed using the MATLAB platform Version 6 (MathWorks, Inc., Natick, MA USA).

### Dose Superposition for Multiple Dwell Positions

In this section, we describe how to calculate multiple dwell doses (D) in the Cartesian voxel coordinate using TG‐43 doses ($D_{e}$), originally developed for a single dwell position in Polar coordinates. In Fig. 2, $D_{e}$ at a point p represents a TG‐43 dose delivered by a stationary source located at the origin. By assuming that a stepping source of each dwell position is located at the origin, this elementary dose from the nth dwell position is calculated by Eq. (1):

(1) $$D_{e}(r_{n},\theta_{n})=S_{k}\cdot \Lambda \cdot \frac{G(r_{n},\theta_{n})}{G(r_{0},\theta_{0})}\cdot F(r_{n},\theta_{n})\cdot g(r_{n}),$$

where $S_{k}=\text{air kerma strength}[U=\;\text{mGy}\;m^{2}\;h^{1}]$, L=dose rate constant[cGy/h/U] at the reference point of $(r_{0},q),r_{0}=1.0\;\text{cm}$ and q0=90°, G(r,q) = geometry factor resulting from spatial distribution of the radioactivity within the line source, g(r) = radial dose function along the transaxial plane, and F(r,q) = anisotropy function describing the dose variation vs angle at an arbitrary point (r,q) in Polar coordinates. With a set of weights for 1 dwell positions, $w_{n}=1\ldots 1$, the total dose to p(r, q), D(p), is a summation of weighted elementary doses, which is expressed by Eq. (2):

(2) $$D(r,\theta )=\sum_{n-1}^{l}w_{n}\cdot D_{e}(r_{n},\theta_{n}).$$

Because our spatial resolution in both y and z directions is Δy=Δz=0.125cm, $r_{n}$ and $\theta_{\mathrm{03B8}}$ are calculated by Eqs. (3) and (4), respectively:

(3) $$r_{n}=\sqrt{{\left(\Delta y\cdot i\right)}^{2}+{\left(\Delta z\cdot j\right)}^{2}}$$

(4) $$\begin{array}{rl} \theta_{n} & =\tan^{-1}(\frac{\Delta y\cdot i}{\Delta z\cdot j})\;\;j\geq \frac{L/2}{\Delta z}\\ & =\frac{\pi }{2}\;\;\;\;\;j=0\\ & =\pi -tan^{-1}(\frac{\Delta y\cdot i}{\Delta z\cdot -j})\;j\leq \frac{-L/2}{\Delta z}, \end{array}$$

where L is the active length of source. Note that the transaxial index i varies from 0 to positive (${}^{+}y$), while the axial index j ranges from negative (distal end, ‐z) through 0 to positive (proximal end, ${}^{+}z$). Finally, for the source movement, D1=0.25cm, the total dose to p(i, j) is a summation of weighted elementary doses given by Eq. (5):

(5) $$D(i,j)=\sum_{n=1}^{l}w_{n}\cdot D_{e}(i,j+l+1-\frac{\Delta z}{\Delta l}\cdot n).$$

### Clinical Feasibility

The potential clinical performance of our optimization method was tested by retrospectively computing dose volume histograms for patients previously treated with balloon brachytherapy. All patients were on a protocol which required consent to use their data for research purposes. Balloon contours and the location of the treatment catheter within the balloon were digitized on all available slices of each patient. From these, balloon volumes, diameters, and DVHs were computed using the appropriate treatment planning systems. For patients treated with the microSelectron HDR system, computations were done with the Plato treatment planning system (TPS), whereas, for patients treated with the Varisource system, Brachy Vision was used. DVH computations were done for the single dwell positions and dwell times that were used in the respective treatments, as well as for the multiple dwell positions that would have been required by our optimization method. In each case, the dose was prescribed at the circle located in the equatorial plane of the balloon, 1 cm from the balloon surface.

## III. RESULTS

**Figure 4:**
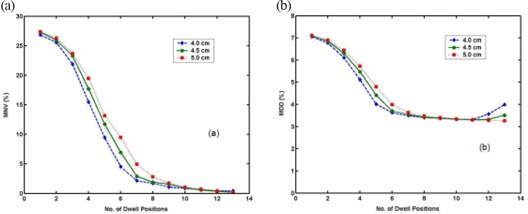
MNV‐optimization parameter (a) and MDD‐optimization parameter (b) vs number of dwell positions for microSelectron. (The legend identifies the diameters of balloons.)

**Figure 5:**
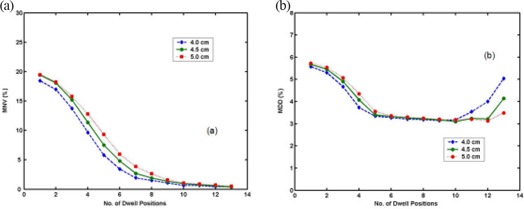
MNV‐optimization parameter (a) and MDD‐optimization parameter (b) vs number of dwell positions for Varisource. (The legend identifies the diameters of balloons.)

Figure 4 shows the objective functions, MNV (a) and MDD (b), vs number of dwell positions for the microSelectron case. Both functions were calculated at optimal weights for each number of dwell positions. For the smaller balloons, the asymptotic saturation of both objective functions starts at a relatively small number of dwell positions. The saturation of both MNV and MDD starts at 6–7 dwell positions. After seven dwell positions, the reduction of both objective functions is minuscule. Therefore, we concluded that dose optimization for the microSelectron case occurred at 7–11 dwell positions with optimal weights (e.g., Table 1 given for 11 dwell positions). The same applied for Varisource. As shown in Fig. 5, the trends of the objective functions for Varisource are similar to those in Fig. 4, and the dose optimization for Varisource also occurred at 7–11 dwell positions with optimal weights (e.g., Table 2 given for 11 dwell positions). Varisource yielded slightly better optimization results for a single dwell position or relatively few dwell positions than microSelectron. However, their optimization at 11 dwell positions achieved almost the same values of both objective functions. At dwell positions >11, the MDD objective functions for both sources begin to increase. This deviation is more pronounced for a balloon of smaller diameter.

**Table 1:** Optimal weights (%) for microSelectron.

*Optimizer*	*Balloon*	*w1*	*w2*	*w3*	*w4*	*w5*	*w6*	*w7*	*w8*	*w9*	*w10*	*w11*
MNV	4.0 cm	11.7	0.5	7.8	14.2	14.8	11.7	7.7	15.2	7.8	2.4	6.3
	4.5 cm	14.5	5.6	5.2	6.8	12.9	15.4	14.2	2.5	8.5	4.8	9.7
	5.0 cm	19.3	1.0	6.7	13.4	11.5	10.0	9.9	2.8	4.6	8.7	12.2
MDD	4.0 cm	5.6	1.3	3.1	15.0	18.9	19.2	16.4	2.9	14.7	2.2	0.6
	4.5 cm	8.0	2.9	0.1	14.5	17.1	18.4	11.5	17.3	4.7	4.9	0.6
	5.0 cm	11.2	4.4	3.1	1.9	14.8	20.2	18.2	14.9	4.8	6.3	0.4

The most distal dwell position is w1, while the most proximal is w11.

**Table 2:** Optimal weights (%) for Varisource.

*Optimizer*	*Balloon*	*w1*	*w2*	*w3*	*w4*	*w5*	*w6*	*w7*	*w8*	*w9*	*w10*	*w11*
MNV	4.0 cm	11.1	5.7	7.8	1.0	12.2	17.7	28.2	5.8	1.0	2.3	7.4
	4.5 cm	12.5	11.6	4.2	4.3	12.1	14.8	11.2	11.5	0.4	11.7	5.6
	5.0 cm	24.4	5.6	0.0	2.3	5.2	10.0	20.5	12.6	5.4	6.9	7.1
MDD	4.0 cm	3.1	0.6	1.8	21.4	20.9	18.4	6.1	21.1	1.1	2.8	2.9
	4.5 cm	7.1	0.2	6.4	1.6	23.5	25.8	12.6	9.2	10.2	1.2	2.3
	5.0 cm	1.8	7.7	5.9	12.1	9.2	17.2	19.9	18.4	2.0	2.8	3.1

The most distal dwell position is w1, while the most proximal is w11.

**Figure 6:**
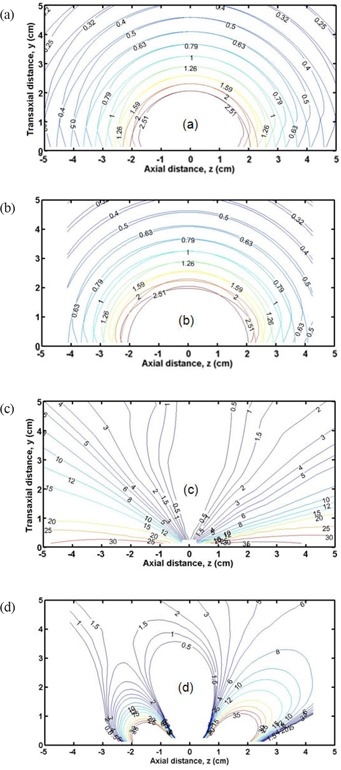
Isodose curves for the microSelectron case normalized to dose at y=3.25cm and z=0 (prescription circle for 4.5 cm ‐diameter balloon) for a single dwell position (a) and for optimized 11 dwell positions (b). Ideal spherical isodose curves are plotted for comparison. Dose‐difference curves for microSelectron are expressed as % deviations from spherical isodoses for a single dwell position (c) and MNV‐optimized 11 dwell positions (d).

Figure 6 shows microSelectron isodose curves for a single dwell position (a) and for eleven dwell positions with MNV optimized weights (b), together with ideal spherical isodose curves for comparison. The isodose curves were normalized to the dose at y=3.25cm and z=0, which corresponds to the prescription line for a 4.5 cm‐diameter balloon. Figure 6 also illustrates % dose difference curves for a single dwell position (c) and for 11 dwell positions (d). The % dose difference is defined as:

(6) $$dose\;difference(\%)=100\times \frac{spherical\;dose-real\;dose}{spherical\;dose}.$$

**Figure 7:**
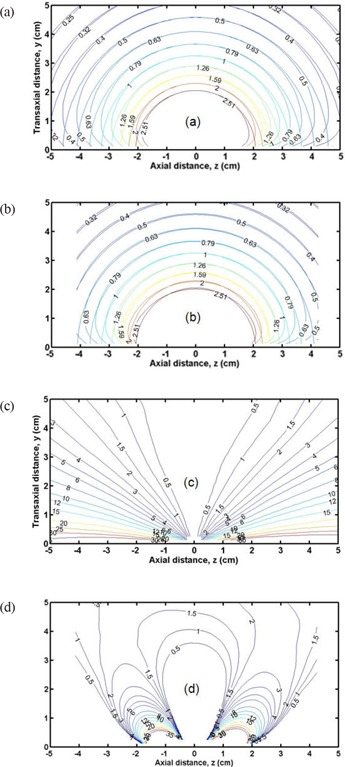
Isodose curves for the Varisource case normalized to dose at y=3.25cm and z=0 (prescription circle for 4.5 cm ‐diameter balloon) for a single dwell position (a) and for optimized 11 dwell positions (b). Ideal spherical isodose curves are plotted for comparison. Dose‐difference curves for Varisource are expressed as % deviations from spherical isodoses for a single dwell position (c) and MDD‐optimized 11 dwell positions (d).

Without optimization (single dwell), a considerable portion of the PTV exceeds 10% difference (see Fig. 6 (c)). However, optimization substantially reduces such dose differences (Fig. 6 (d)). In addition, there is potential skin sparing (approx. 4%) in the region of −1<z<1cm and y<2.5cm if the skin is approximately tangent to the source movement. Figure 7 shows the similar isodose curves for the Varisource case for a single dwell position [(a) and (c)], and for 11 dwell positions [(b) and (d)] with MDD optimized weights.

**Figure 8:**
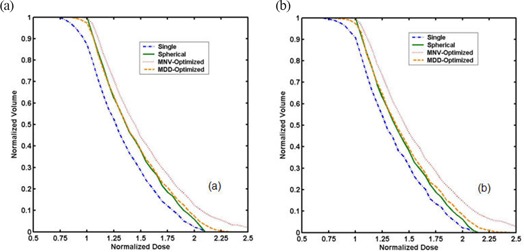
PTV‐dose volume histograms (DVH) for a 4.5 cm diameter balloon for the microSelectron (a) and Varisource (b) cases. (The legend identifies the Dvh for a single dwell position, an isotropic source, MNV‐optimized 11 dwell positions, and MDD‐optimized 11 dwell positions.)

Figure 8 shows dose volume histograms (DVH) for the PTV of a 4.5 cm diameter balloon for microSelectron (a) and Varisource (b) cases. When a single dwell position is used, 11%‐13% of the PTV, mostly the distal part of the PTV, does not receive the prescription dose. The solid lines are the DVH of the ideal spherical dosimetry. The MNV‐optimizer (dotted‐line) is designed to give best coverage of PTV‐voxels, while the MDD‐optimizer (dashed‐line) attempts to match the actual doses to the spherical doses. Thus, the DVH of the MNV‐optimized dosimetry shows a perfect PTV coverage, but 11% of the PTV receives more than twice the prescription dose, whereas in the ideal spherical case, only 5% of the PTV receives such high doses. The DVH of the MDD‐optimized dosimetry shows a very close match to the solid lines (spherical), but about 2%‐3% of the PTV receives less than the prescription dose. Tables 1 and 2 list the MNV‐ and MDD‐optimized weights (%) of 11 dwell positions for the two sources. Each table includes data for the three balloons of different diameters optimized by the MNV and MDD methods. The 1st weight (w 1) corresponds to the most distal dwell location, while the 11th weight (w11) corresponds to the most proximal dwell location. The 6th dwell position is in the center of the balloon.

We also verified clinical practicability and reproducibility of the optimization schemes developed in this study. The two commercial treatment planning systems were used to generate the optimal conformal plans of nine patients. The analyses of the plans are summarized in Table 3, including patient's label and balloon diameter, the PTV coverage by a single dwell position and optimized 11 dwell positions, and HDR system. It is noteworthy that although all the patients were treated with spherical balloons, the diameters measured on patient CT‐images varied within a certain range (see parenthesis in the 2nd column of Table 3). Overall, the treatment plans optimized by MNV and MDD achieved over 97.5% and 94.3% of the PTV coverage, respectively, while those of a single dwell position showed the PTV underdosage of 10%–17%. Note that since patient balloons inserted into the surgical bed are not perfectly spherical, the PTV coverage of optimized plans did not achieve the results obtained in the idealized case described in Fig. 8.

**Table 3:** PTV prescription dose coverage with and without the optimization for patients with balloons of various diameters. They were obtained by using commercial treatment planning systems and the optimal weights of Tables 1 and 2. The patient's balloon was not a perfect sphere, and thus the variations of diameters measured at various directions were indicated in parentheses of the 2nd column.

*Patients*	*Balloon diam. (cm)*	*Single dwell position (%)*	*11 dwell positions (%)*		*Source/TPS*
			*MNV*	*MDD*	
1	4.3 (4.25–4.3)	89.9	97.6	96.6	Nuc/Plato^a^
2	4.6 (4.4–4.7)	86.7	97.6	96.2	Nuc/Plato
3	4.8 (4.7–4.8)	90.8	97.6	96.6	Nuc/Plato
4	3.8 (3.7–3.85)	88.1	99.5	97.2	Nuc/Plato
5	4.0 (3.9–4.1)	89.6	97.8	94.3	Nuc/Plato
6	5.0 (5.3–5.7)	87.4	98.8	96.7	Var/Brachy^b^
7	5.0 (5.1–5.2)	89.2	98.3	96.9	Var/Brachy
8	4.3 (4.2–4.4)	81.5	97.6	95.9	Var/Brachy
9	4.3 (4.2–4.4)	83.2	99.2	97.1	Var/Brachy

a Nucletron Plato Treatment Planning System.

b Varian BrachyVision Treatment Planning System.

## IV. DISCUSSION AND CONCLUSIONS

Our volumetric dose optimization showed a close PTV‐DVH agreement with the results obtained using linear or multiple‐points optimization in the literature.^(7)^ ^,^ ^(8)^ ^,^ ^(11)^ All of these suggest that 11%–13% of the PTV does not receive the prescription dose, primarily due to anisotropy, and propose multiple dwell positions for an improved PTV coverage. As shown in Fig. 8, the shape of the MDD‐optimized DVH curve is very similar to that of the MNV‐optimized DVH curve. Therefore, by increasing the total dwell times calculated with the MDD‐optimizer by 2%–3% using the same weights, one can also achieve complete PTV‐coverage. This PTV coverage is similar to that achieved by the MNV‐optimizer, with a penalty of 2%–3% increase in skin dose. The choice of either MNV or MDD optimizer might depend on the relative importance of skin toxicity and the interface between the balloon and tissue. In an earlier study,^(9)^ we reported that the entire PTV is underdosed by 4%–10% due to the lack of scattering medium in the breast and the attenuation by contrast medium in the balloon. In this case, MNV‐optimized dosimetry might be better than the MDD‐optimizer, since it still provides at least 97% PTV coverage at 105% of the prescription dose (i.e. at 1.05 times the normalized dose in Fig. 8).

When the skin sparing constraint is applied to the MDD‐optimizer, the results showed that the average dose to assigned skin‐voxels was almost the same (within approx. 2% difference) as that without the skin sparing constraint. However, the PTV coverage with the skin sparing constraint was slightly worse (only in a small portion of the PTV closest to the skin) than that without the skin sparing constraint. Interestingly, even without applying the skin sparing constraint, some skin sparing was already achieved because of ellipsoidal isodose distributions of optimized dosimetry along the source axis if the skin is approximately tangent to the source axis). This is illustrated in Figs. 6 (d) and 7 (d) where differences of a few percent were found in the region of z=−1cm to1cm and y<2.5cm. On the contrary, the skin dose for a stationary source (single dwell position) increases by over 10% when one attempts, by increasing the dwell time, to achieve the same level of the PTV coverage as observed in the MNV‐ and MDD‐optimizers.

In conclusion, this study presents a methodology for optimization schemes for many possible clinical situations, using two Ir192 HDR sources and spherical balloons of 4–5 cm diameters currently available for breast balloon brachytherapy. The method can be easily implemented clinically. A new optimization scheme for ellipsoidal balloons recently available is under investigation.

## ACKNOWLEDGEMENTS

This study was in part supported by the Long‐Term Nuclear Development Program from the Korean Ministry of Education, Science and Technology.
